# Enhanced Synthesis of Carbon Nanomaterials Using Acoustically Excited Methane Diffusion Flames

**DOI:** 10.3390/ma8084805

**Published:** 2015-07-29

**Authors:** Shuhn-Shyurng Hou, Kuan-Ming Chen, Zong-Yun Yang, Ta-Hui Lin

**Affiliations:** 1 Department of Mechanical Engineering, Kun Shan University, Tainan 71070, Taiwan; E-Mails: acntbergo739@gmail.com (K.-M.C.); zongyun815@gmail.com (Z.-Y.Y.); 2 Research Center for Energy Technology and Strategy, National Cheng Kung University, Tainan 70101, Taiwan; 3 Department of Mechanical Engineering, National Cheng Kung University, Tainan 70101, Taiwan

**Keywords:** flame synthesis, carbon nanotubes, carbon nano-onions, acoustic excitation

## Abstract

Acoustically modulated methane jet diffusion flames were used to enhance carbon nanostructure synthesis. A catalytic nickel substrate was employed to collect the deposit materials at sampling position *z* = 10 mm above the burner exit. The fabrication of carbon nano-onions (CNOs) and carbon nanotubes (CNTs) was significantly enhanced by acoustic excitation at frequencies near the natural flickering frequency (ƒ = 20 Hz) and near the acoustically resonant frequency (ƒ = 90 Hz), respectively. At these characteristic frequencies, flow mixing was markedly enhanced by acoustic excitation, and a flame structure with a bright slender core flame was generated, which provided a favorable flame environment for the growth of carbon nanomaterials. The production rate of CNOs was high at 20 Hz (near the natural flickering frequency), at which the gas temperature was about 680 °C. Additionally, a quantity of CNTs was obtained at 70–95 Hz, near the acoustically resonant frequency, at which the gas temperature was between 665 and 830 °C. However, no carbon nanomaterials were synthesized at other frequencies. The enhanced synthesis of CNOs and CNTs is attributed to the strong mixing of the fuel and oxidizer due to the acoustic excitation at resonant frequencies.

## 1. Introduction

Carbon nanotubes (CNTs) have excellent mechanical, thermal, optical, and electrical properties [1,2]. They have been used for many electronic, optical, and magnetic applications, such as gas sensors [3], high-temperature superconductors [4], scanning microscope tips [5], and hydrogen storage media [6]. Many methods have been proposed for the synthesis of CNTs, including arc discharge [7], laser ablation [8], spray pyrolysis [9], and chemical vapor deposition (CVD) [10]. However, most of these methods are expensive and complex. Recently, flame synthesis of carbon nanomaterials has been widely explored due to its advantages over other production methods that use expensive electricity as the heat source. It has been shown that flame synthesis makes possible the low-cost mass production of CNTs [11].

Nakazawa *et al.* [6] investigated premixed flame synthesis of CNTs in a wall stagnation flow. Nitrogen was used to dilute a rich ethylene/air premixed gas. Increasing the exposure time of the catalyst surface to the flame increased the number of layers in the obtained CNTs. It was observed that on appropriate portions of the wall (catalyst) surface, abundant multi-walled carbon nanotubes (MWCNTs) with diameters of 8–21 nm were synthesized. 

Hu *et al.* [12] examined the dominant factors governing the fabrication of CNTs and carbon nano-onions (CNOs) using counter-flow diffusion flames. They focused on the influence of the CH_4_/C_2_H_4_ ratio on the fuel side and oxygen concentration on the oxidizer side. The results showed that the synthesis of CNTs significantly depended on the oxygen concentration. For oxygen concentrations other than 30%, the fuel composition had only a minor effect on the morphologies of the synthesized carbon nanomaterials.

Chung *et al.* [13] used ethylene jet diffusion flames modulated by acoustic excitation to fabricate CNOs on a catalytic nickel substrate. It was found that the rate of yield of CNOs was high at frequencies near the natural flickering frequency (10–30 Hz) for the axial position *z* = 10 mm and a gas temperature in the range of 420–500 °C. Additionally, for both *z* = 5 and 10 mm, CNOs were obtained at frequencies near the acoustically resonant frequency (60–70 Hz), at which the gas temperature was in the range of 620–720 °C.

In Chung *et al.*’s study [13], only CNOs were synthesized when ethylene diffusion flames under the influence of acoustic excitation were used. It has been reported that carbon sources strongly impact the synthesis of carbon nanomaterials [14]. Therefore, in the present study, methane was used instead of ethylene and the results were compared to those of Chung *et al.* [13] to determine the influence of fuel structure on the synthesis of carbon nanostructures.

Experiments were performed to examine the effect of the acoustic excitation frequency on the yield and structure of carbon nanomaterials synthesized in laminar methane diffusion flames.

## 2. Experiments

**Figure 1:**
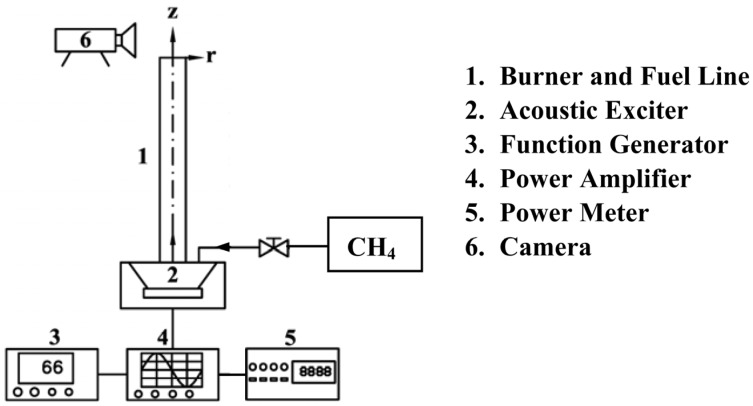
Schematic of jet flow system with acoustic modulation.

Figure 1 schematically shows the experimental system. The simple round-jet burner was comprised of a single stainless steel tube with inner and outer diameters of 1.1 and 1.3 cm, respectively. The burner tube was 50 cm in length, which was sufficiently long to produce a fully developed laminar velocity profile at the exit. Methane (CH_4_, 99.9 wt%), utilized as the fuel, was controlled by a conventional rotameter, and introduced into the forcing chamber, in which the acoustic exciter was installed. The methane passed through the burner tube and flowed into the ambient air. A periodically excited jet flame was established at the burner exit by a jet of methane mixed with ambient air. The mean velocity of the fuel jet was kept at *V* = 0.15 ms^−1^ during the experiment.

The acoustic modulation system consisted of a power amplifier, a power meter, a function generator, an acoustic exciter (an 80-W, 8-inch-diameter (20.32 cm) loudspeaker), and a fuel line. The total length of the fuel line from the acoustic exciter to the burner exit was 2.5 m. The acoustic exciter was installed coaxially beneath the fuel line in the forcing chamber (made of hermetical acrylics; 24 cm × 24 cm × 13 cm), which was supplied with a sinusoidal driving current of adjustable frequency in the range of 0 (unexcited) to 3000 Hz using the function generator. The acoustic amplitude and frequency were well controlled by the function generator, power amplifier, and power meter. Amplitude is directly related to the acoustic energy or intensity of sound. The power of the acoustic exciter, *P*, which is related to the amplitude of the excitation, was adjustable, but was kept at 5 W in the experiment.

**Figure 2:**
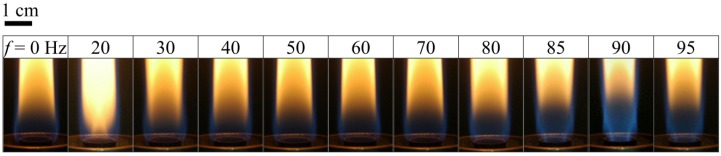
Flame structures for various modulation frequencies at *V* = 0.15 ms^−1^ and *P* = 5 W.

The excitation frequency and amplitude could be tuned to produce various flame shapes. The flame appearance (see Figure 2, discussed later) significantly changed in the region near the nozzle exit (flame base) when various frequencies were applied at a fixed power of 5 W (corresponding to a constant amplitude). Hence, the observation of flame shapes was focused in the region 0–2 cm above the burner exit. The flame behavior of resonant and non-resonant cases was compared.

The camera system included a digital single-lens reflex camera (Nikon D70, Tokyo, Japan), a high-speed camera (Kodak Motion Corder Analyzer, Model SR series, San Diego, CA, USA), and image analysis software (Uthscsa Image Tool Version 3, Microsoft, Redmond, WA, USA). The camera system was used to capture the time-averaged and instantaneous images of the flame length and structure. The temperature distribution along the axis of symmetry of the burner tube was measured using an R-type thermocouple (Pt/Pt-13% Rh and 0.05 inch (1.27 mm) diameter) driven by a three-dimensional (3D) positioner. For the synthesis of carbon nanomaterials, the substrate was moved using a 3D positioner. The sampling position (*z*) was chosen to be 10 mm above the burner exit plane along the axis (*r* = 0) where the acoustic effect was strong. The deposition time was kept at 120 s. The deposited materials were characterized via field-emission scanning electron microscopy (FE-SEM, JEOL JSM-7000F, Tokyo, Japan) and high-resolution transmission electron microscopy (HR-TEM, JEOL JEM-2100, Tokyo, Japan). Moreover, Raman scattering spectra were obtained with a JASCO Ventuno-21 micro-Raman spectrophotometer (Jasco Co., Tokyo, Japan) using an Ar-ion laser with a frequency of 532 nm at room temperature to identify the structure of products.

The acoustically resonant frequency of the methane flame was calculated as [15]:

(1) $$f=n\frac{a}{2L}$$

where *a* is the speed of sound in tested gas (~446 ms^−1^ for methane); *L* is the length of the fuel line (2.5 m); and *n* is the frequency mode (*n* = 1 in the present study). Hence, the first acoustically resonant frequency of methane is 90 Hz.

## 3. Results and Discussion

### 3.1. Flame Parameters

**Figure 3:**
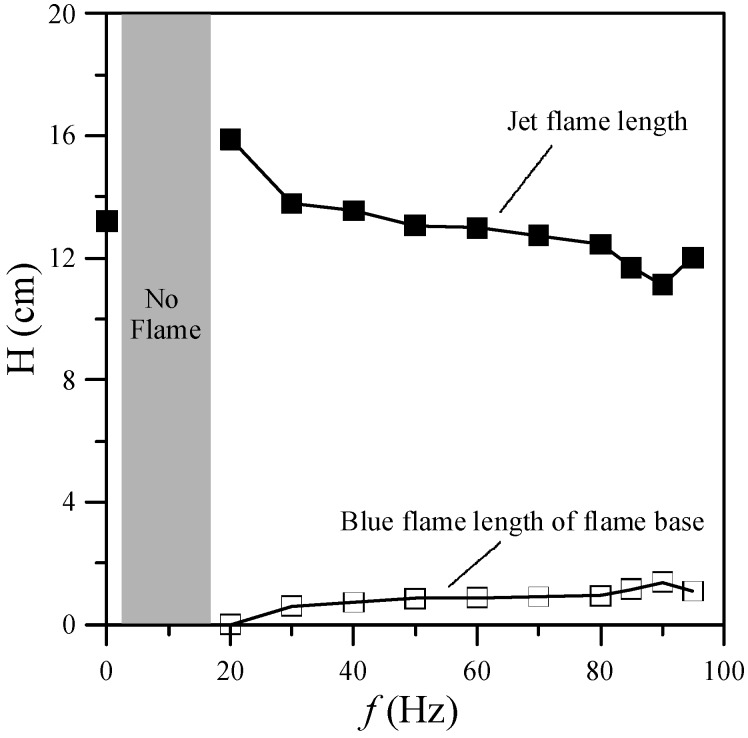
Flame lengths and blue flame lengths of flame base at various excitation frequencies.

Figure 2 shows images of the flame structure at various excitation frequencies (*f*) in the range of 0–95 Hz. Figure 3 shows the jet flame length and blue flame length of the flame base for various excitation frequencies. In general, the low-frequency oscillation (10–15 Hz) or “flickering” of laminar diffusion flames is caused by buoyancy effects induced by Kelvin-Helmholtz instability [16]. As shown in Figure 2, the image for *f* = 0 Hz (without acoustic excitation) shows a typical laminar jet flame, which has a single-flame structure. When the excitation frequency was close to the flame flickering frequency (20 Hz), the flame was composed of a slender yellow central flame and a blue outer flame. When the excitation frequency was increased from 20 to 80 Hz, the radius of the central flame in the region near the burner exit increased gradually and the flame color became less bright since the frequency deviated from the natural flickering frequency. Moreover, the jet flame length decreased, whereas the blue flame length of the flame base increased, as shown in Figure 3. When the excitation frequency was increased from 80 to 90 Hz, the radius of the core flame gradually decreased. Moreover, the flame length decreased and the blue flame length of the flame base increased.

Figure 2 also shows that the shortest flame length and the longest blue flame length of the flame base occurred at the acoustically resonant frequency (90 Hz). As shown in Figure 2, when the excitation frequency approached the acoustically resonant frequency (90 Hz), the flame had a double-flame structure that consisted of a blue central flame and a blue outer flame (e.g., 85 and 90 Hz). The central flame color became brighter when the excitation frequency was increased from 80 to 90 Hz. At the first acoustically resonant frequency, 90 Hz, the flame structure showed the slenderest and most luminous core flame with the broadest blue outer flame. It is noted that the acoustic excitation caused the reverse flow to induce strong air entrainment, which in turn enhanced flow mixing, slenderizing the blue core flame and leading to the partially premixed blue outer flame. Note that at the natural flickering frequency (*f* = 20 Hz), the central flame was yellow, whereas at the acoustically resonant frequency (*f* = 90 Hz), the central flame was blue. Under these acoustic excitation conditions, a significant amount of air was sucked into the burner exit during the entrainment part of the cycle. Consequently, the jet flow was compressed and the central flame burned more brightly [13,17]. These characteristics imply that the effect of air entrainment at the natural flickering frequency (*f* = 20 Hz) was slightly weaker than that at the acoustically resonant frequency (*f* = 90 Hz).

There are two modes of resonance associated with acoustic excitation, namely natural flickering and acoustical resonance [13]. Natural flickering occurred for acoustic excitation frequencies in the range of 10–20 Hz due to the interaction between the hot combustion gas and the ambient air, which induced large vortices. At acoustical resonance, a large amount of ambient air was entrained into the burner exit and mixed with the fuel. From Equation (1), the resonant frequency is about 90 Hz for methane. The change in the acoustically resonant frequency resulted in a significant difference in the flame structure and appearance, which in turn significantly influenced the formation of carbon nanostructures.

The flame structures shown in Figure 2 can be classified into three main types, namely natural flickering, non-resonant, and acoustically resonant. At the acoustically resonant frequency, a double-layer flame structure was observed. Both the natural flickering mode and the acoustically resonant mode resulted in a flame with a luminous core, which was carbon-rich and had a high temperature. Furthermore, it is expected that the suitable range of fuel concentration for synthesis was wider at these two frequencies than at other frequencies [13].

**Figure 4:**
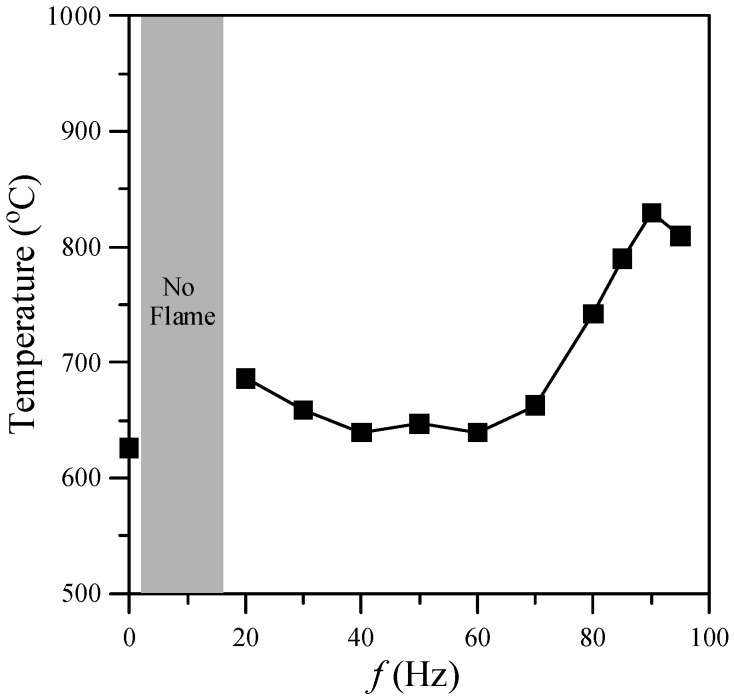
Gas temperature at *z* = 10 mm above burner exit for various excitation frequencies.

One of the important factors in nanostructure synthesis is the temperature of the environment. This investigation focused on the region near the flame base, and hence the mean temperatures (*T*) at *z* = 10 mm above the burner exit for acoustic excitation frequencies of 0–95 Hz were examined. The results are presented in Figure 4. As shown, the gas temperature of the flame with acoustic excitation was higher than that without acoustic excitation due to a more slender core flame (*i.e.*, higher temperature gradient). Two temperature peaks, at the natural flickering and acoustically resonant frequencies, respectively, were observed as the excitation frequency increased. The first peak (680 °C) appeared at *f* = 20 Hz and the second peak (830 °C) appeared at the acoustically resonant frequency (*f* = 90 Hz).

### 3.2. Flame Synthesis of Carbon Nanomaterials

The focus of this investigation was analyzing the formation of carbon nanomaterials sampled at *z* = 10 mm above the burner exit along the centerline for a fixed power output of 5 W. This was done because the flow field near the flame base (0 ≤ *z* ≤ 15 mm above the burner exit) was strongly affected by acoustic excitation. In the experiments, SEM was employed to analyze the fabrication of carbon nanomaterials. The formation of carbon nanomaterials was greatly affected by acoustic excitation at frequencies near the natural flickering frequency and the acoustically resonant frequency.

**Figure 5:**
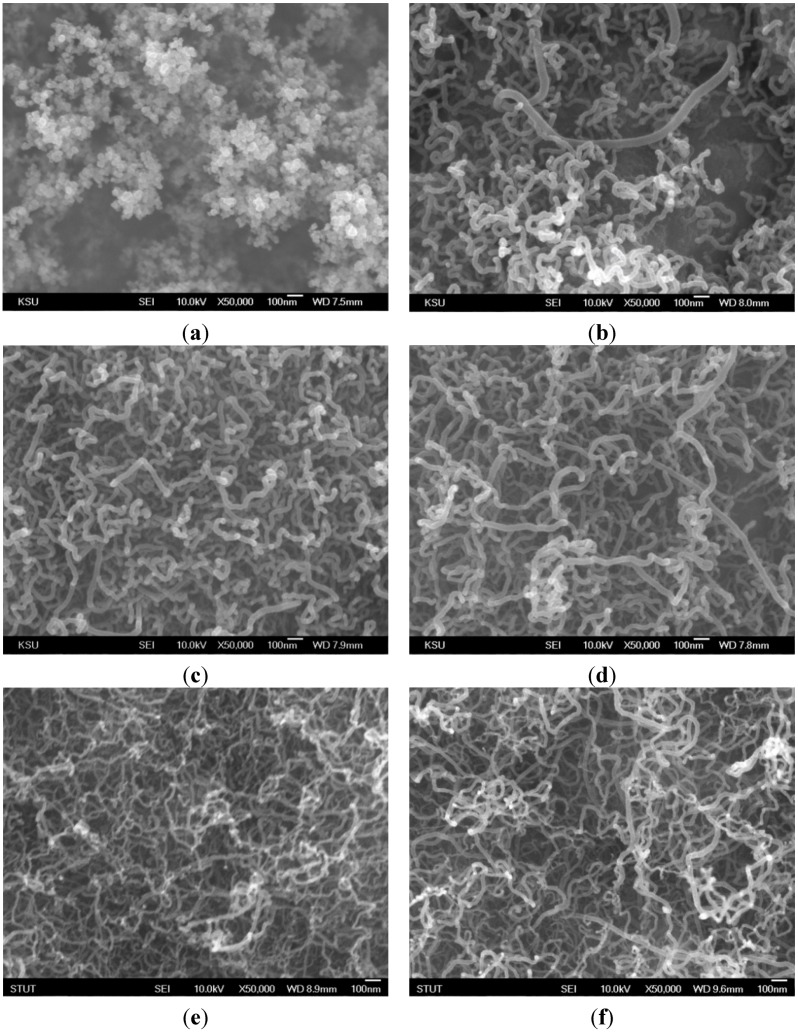
SEM images of carbon nanomaterials synthesized at *z* = 10 mm for *f* values of (**a**) 20; (**b**) 70; (**c**) 80; (**d**) 85; (**e**) 90; and (**f**) 95 Hz.

As shown in Figure 5, there was a large quantity of carbon nanomaterials (piled like bunches of grapes) on the substrate at frequencies near the natural flickering frequency (*f* = 20 Hz). The SEM image shows a CNO microstructure. Additionally, a quantity of CNTs was obtained at 70–95 Hz, near the acoustically resonant frequency, at which the gas temperature was 665–830 °C. However, almost no CNOs or CNTs were synthesized at other frequencies due to low temperature or a lack of carbon sources. The enhanced synthesis of carbon nanomaterials was attributed to strong mixing of the fuel and oxidizer due to acoustic excitation at the resonant frequencies. The results verify that the main products were CNOs and CNTs. In Chung *et al.* [13], ethylene was used as the fuel, and only CNOs were fabricated. In this study, methane was employed as the fuel. Only CNOs were synthesized at the natural flickering frequency, and only CNTs were grown at the acoustically resonant frequency. In Chung *et al.* [13], a yellow core flame appeared at both the natural flickering frequency and the acoustically resonant frequency. In contrast, in the present study, yellow and blue core flames were observed at the natural flickering frequency and the acoustically resonant frequency, respectively. The flame structure affected the formation of carbon nanomaterials. It is noteworthy that CNOs were synthesized in a sooty yellow core flame, whereas CNTs were fabricated in a blue core flame. The slender and luminous core flame led to good synthesis of carbon nanostructures due to a favorable environment temperature and a high carbon precursor concentration.

**Figure 6:**
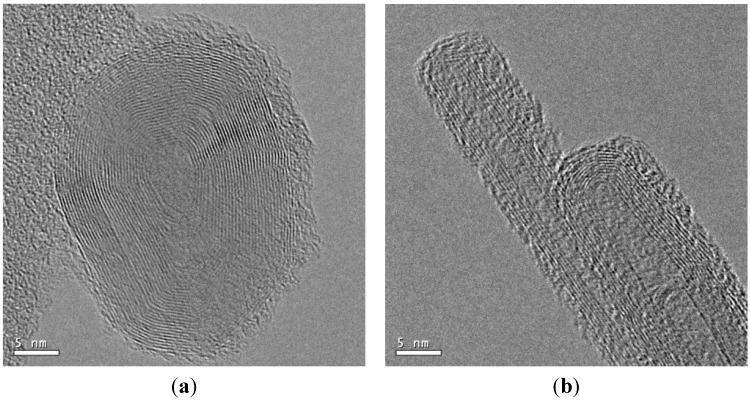

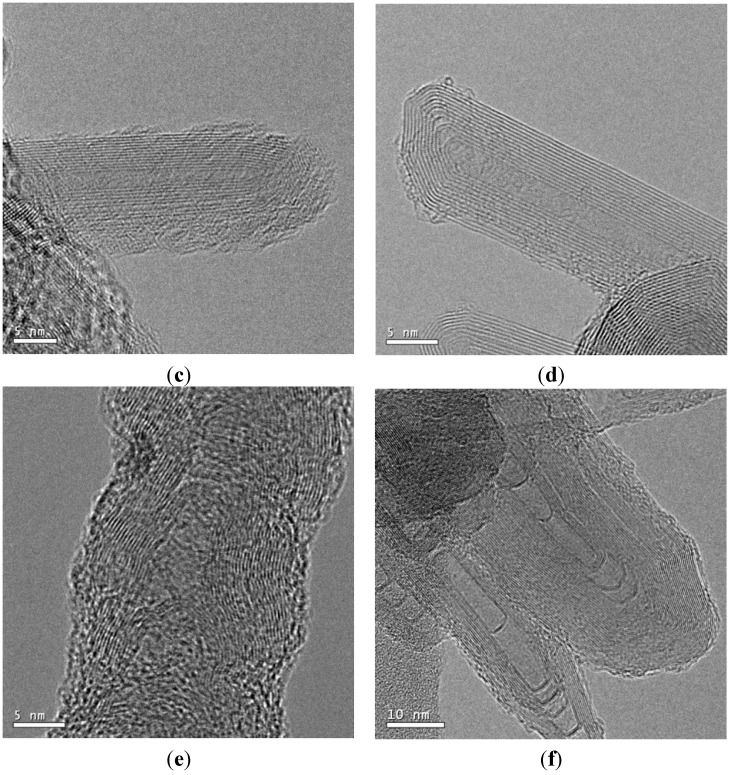
TEM images of carbon nanomaterials synthesized at *z* = 10 mm for *f* values of (**a**) 20; (**b**) 70; (**c**) 80; (**d**) 85; (**e**) 90; and (**f**) 95 Hz.

**Figure 7:**
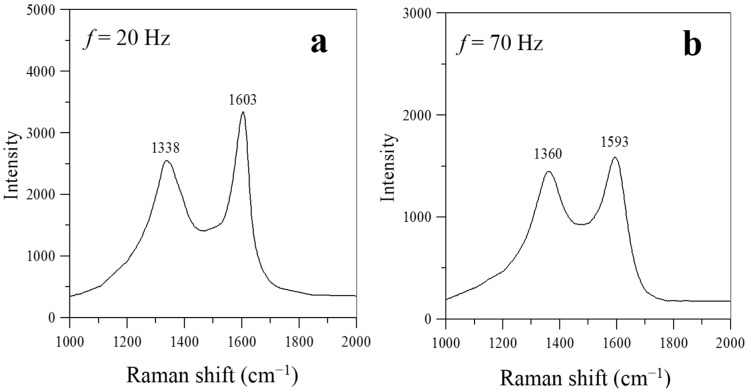

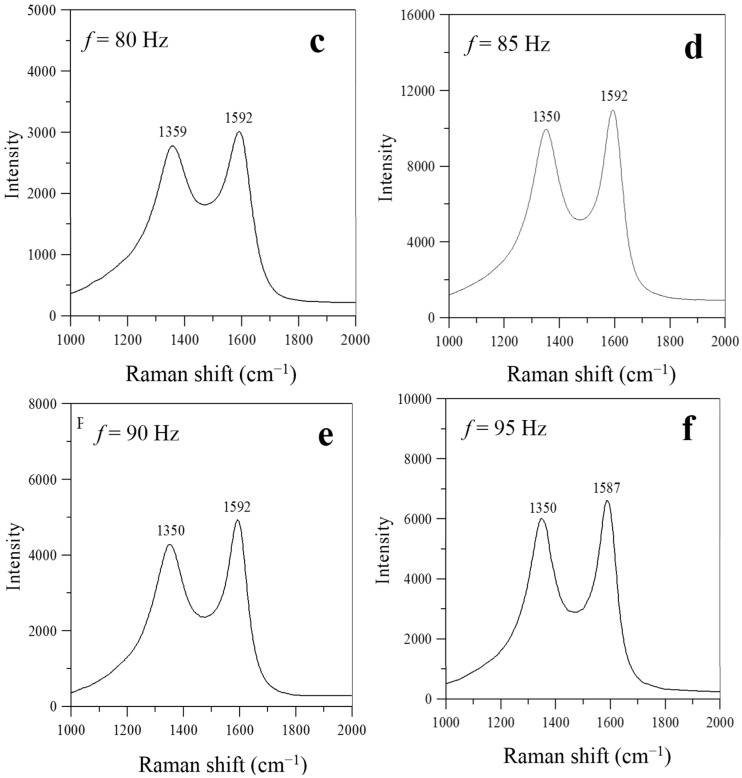
Raman spectra of carbon nanomaterials synthesized at *z* = 10 mm and *P* = 5 W for *f* values of (**a**) 20; (**b**) 70; (**c**) 80; (**d**) 85; (**e**) 90; and (**f**) 95 Hz.

Figure 6 shows TEM images of carbon nanomaterials synthesized at *z* = 10 mm for *f* = 20, 70, 80, 85, 90, and 95 Hz. Figure 7 shows Raman spectra of carbon nanomaterials synthesized at *z* = 10 mm for *f* = 20, 70, 80, 85, 90, and 95 Hz. As shown in Figure 6a, a typical spherical CNO, which consists of a concentric arrangement of carbon layers, with a diameter of about 30 nm formed at *f* = 20 Hz. It had a hollow core wrapped in layers of well-crystallized graphitic sheets (32 layers). Similarly, the inter-layer spacing of the CNO was about 0.34 nm, which is consistent with the Raman spectrum (shown in Figure 7a). Two different types of CNT were found in the deposit materials on the Ni substrate. Figure 6c,d show HR-TEM images of CNTs with a straight and tubular structure, and Figure 6e,f show CNTs with a bamboo-like structure. Figure 6b shows that tubular and bamboo-like structures can co-exist. It has been suggested that spherical catalyst particles catalyze the formation of straight and tubular CNTs without any internal compartment caps, whereas non-spherical catalyst particles tend to form bamboo-like CNTs with an internal compartment cap [13,17].

As shown in Figure 7, two prominent peaks, at ~1590 cm^−1^ (G-band) and ~1330 cm^−1^ (D-band), respectively, appear in the Raman spectra at frequencies near the natural flickering frequency (*f* = 20 Hz) or the acoustically resonant frequency (*f* = 70–95 Hz). No prominent peaks of G- and D-bands were observed simultaneously in the Raman spectra for the flame without acoustic excitation (*f* =0 Hz) and for the flames experiencing non-resonant acoustic modulation at frequencies of *f* = 30–60 Hz. This is because nearly no carbon nanostructures were produced under these conditions. Figure 7 also shows that the intensity ratios of the D- and G-bands were lower than one (I_D_/I_G_ < 1) for the flames with acoustic excitation at frequencies close to the natural flickering frequency (*f* = 20 Hz) and near the acoustically resonant frequency (*f* = 70, 80, 85, 90, and 95 Hz). Therefore, the characteristics of the Raman spectra in Figure 7 show that the CNOs synthesized at frequencies near the natural flickering frequency and the CNTs synthesized at frequencies near the acoustically resonant frequency had a high degree of graphitization.

## 4. Conclusions

This study examined the effect of acoustic modulation on the synthesis of carbon nanomaterials in a laminar jet diffusion flame. The results show that a single-layer flame structure was produced without acoustic modulation (*f* = 0 Hz), while a double-layer flame structure was generated for frequencies near the natural flickering frequency (*f* = 20 Hz) and the acoustically resonant frequency (*f* = 90 Hz).

The synthesis of carbon nanomaterials was significantly enhanced by acoustic excitation near the natural flickering frequency and the acoustically resonant frequency. Under these two excitation conditions, a double-layer flame structure formed with a slender core flame and a more luminous outer flame (*i.e.*, higher temperature). This double-layer flame structure provided a favorable flame environment for the fabrication of carbon nanomaterials. It was verified that the flame synthesis of carbon nanomaterials was significantly influenced by acoustic modulation. A large quantity of CNOs formed at *f* = 20 Hz, at which the gas temperature was about 680 °C, and a large quantity of CNTs formed at *f* = 70–95 Hz, at which the gas temperature was 665–830 °C. However, at other frequencies, almost no carbon nanomaterials formed. It is noteworthy that CNOs were synthesized in a sooty yellow core flame. This is consistent with the results of Chung *et al.* [13]. CNTs were fabricated in a blue core flame. Two different types (tubular structure and bamboo-like structure) of CNT were found at frequencies near the acoustically resonant frequency (90 Hz). Moreover, the CNOs synthesized at frequencies near the natural flickering frequency and the CNTs synthesized at frequencies close to the acoustically resonant frequency had a high degree of graphitization since the intensity ratios of the D- and G-bands were lower than one (I_D_/I_G_ < 1).
